# Prevalence, Risk Factors, and Preventive Strategies of Hypertension Among Young Adults in the United Arab Emirates

**DOI:** 10.3390/ijerph23060698

**Published:** 2026-05-25

**Authors:** Aws Raid Hussain Aljubori, Mahmoud Nabil M. Abutartour, Ibrahim Abdulla Darwish Ali, Mohammed Ghaith Al Haj Younes, Jayakumary Muttappallymyalil

**Affiliations:** Community Medicine, College of Medicine, Gulf Medical University, Ajman P.O. Box 4184, United Arab Emirates; 2022md47@mygmu.ac.ae (A.R.H.A.); 2022md89@mygmu.ac.ae (M.N.M.A.); 2022md105@mygmu.ac.ae (I.A.D.A.); 2022md81@mygmu.ac.ae (M.G.A.H.Y.)

**Keywords:** hypertension, prevalence, preventive practices, young adults, UAE

## Abstract

**Highlights:**

**Public health relevance—How does this work relate to a public health issue?**

- Addresses the growing burden of early-onset hypertension among young adults, a key contributor to cardiovascular diseases globally and in the UAE.
- Highlights modifiable lifestyle and behavioral risk factors in a rapidly urbanizing population, aligning with global non-communicable disease prevention priorities.

**Public health significance—Why is this work of significance to public health?**

- Reveals a potentially higher-than-expected prevalence of hypertension in young adults in the UAE, indicating early risk accumulation and future healthcare burden.
- Provides region-specific evidence to support targeted prevention strategies, contributing to global literature on hypertension epidemiology in younger populations.

**Public health implications—What are the key implications or messages for practitioners, policy makers and/or researchers in public health?**

- Emphasizes the need for early screening, lifestyle interventions, and awareness programs targeting young adults within primary healthcare and community settings.
- Supports policy development for integrated NCD prevention strategies, including health promotion, behavioral interventions, and research on culturally appropriate risk reduction approaches.

**Abstract:**

**Background:** Hypertension is one of the most common noncommunicable diseases. **Objectives:** This research assessed the magnitude of hypertension among young adults, identified its key determinants, and explored potential strategies adopted for prevention. **Methods:** A cross-sectional design was employed, including 1606 participants aged 18 years and older, recruited through convenience sampling from universities and community settings. Data were collected using a content-validated questionnaire covering sociodemographic information, personal and family medical history, and lifestyle habits. **Results:** Of the participants, 993 (61.8%) reported hypertension, nearly double previous national estimates. Male gender, age ≥ 30 years, and family history were significant risk factors, along with smoking, alcohol use, sedentary lifestyle, and unhealthy diet, while physical activity and dietary modification were protective. Despite high prevalence, only 22.1% had controlled blood pressure and 17.8% adhered to medication, with 51.5% relying on herbal remedies. **Conclusions:** These findings highlight the urgent need for early screening, youth-focused awareness, and culturally tailored interventions to reduce hypertension and prevent long-term cardiovascular complications. Hypertension among young adults in the UAE is a major public health concern, requiring integrated strategies combining education, lifestyle modification, and medical management to improve outcomes.

## 1. Background

Hypertension, the “silent killer,” is an increasing public health concern among young adults, despite being traditionally associated with older populations. It is defined as persistently elevated blood pressure and is a major risk factor for cardiovascular disease, stroke, and kidney failure [1]. Its often-asymptomatic nature leads to underdiagnosis, highlighting the need for early prevention and awareness.

In Western countries, hypertension is increasingly affecting young adults due to poor diet, sedentary lifestyles, and stress [2]. In the US, about one in three adults has hypertension, including a significant proportion aged 18–39 [2]. Evidence also shows that family history increases risk, highlighting the need for early identification and intervention [3]. The MENA region shows a rising trend of hypertension in young adults, driven by high salt intake, obesity, and sedentary lifestyles. Studies in the UAE and North Africa, including Egypt, report increasing prevalence linked to poor diet and low physical activity. These findings highlight the need for stronger public health campaigns and education [3,4,5].

In the UAE, hypertension is a growing concern among young adults, reporting high rates of pre-hypertension and hypertension among university students, linked to poor diet and low physical activity [5]. Many remain unaware of their condition, increasing future cardiovascular risk. Although public health campaigns have been introduced, gaps in awareness and knowledge persist [6]. Sub-Saharan Africa is also experiencing a rising burden of hypertension among young adults. In Zimbabwe, Sabapathy et al. (2023) reported that 21% of individuals aged 18–35 had high-normal blood pressure or hypertension, reflecting broader regional trends driven by urbanization, poor diet, obesity, and physical inactivity [7]. In Kenya, 18.7% of young adults attending a hospital had elevated blood pressure, with obesity and family history identified as key risk factors [8].

Hypertension in young adults is multifactorial, involving lifestyle and genetic factors. Key risks include obesity, high sodium intake, low potassium intake, physical inactivity, stress, smoking, and alcohol use [9,10,11]. Psychosocial stress and family history further increase susceptibility [11,12]. This study examines the prevalence, risk factors, and awareness of hypertension among young adults to identify gaps in knowledge and prevention, with the aim of informing targeted interventions to reduce long-term cardiovascular and related complications [13,14]. By identifying the key determinants of hypertension in young adults, the findings will help public health officials and healthcare providers design better interventions that are tailored to this age group, fostering healthier lifestyles and early detection.

## 2. Materials and Methods

### 2.1. Study Design, Study Population, Sample Size, Sampling Procedure, and Setting

A community-based cross-sectional study was conducted in the UAE to assess the prevalence of hypertension and associated risk factors among adults aged ≥18 years. The sample size (1600) was calculated using N = 4 PQ/L^2^ with 5% precision and an assumed prevalence of 30%, plus 10% for nonresponse. Participants were recruited through convenience sampling in public and university settings, while individuals with language barriers, cognitive limitations, or unavailability were excluded.

### 2.2. Study Instrument and Validation Procedure

Data were collected using a structured questionnaire developed from literature on prevalence and factors associated with hypertension, lifestyle, and prevention, and validated by two Public Health experts and one physician. Self-reported physicians’ diagnosed hypertension profile was elicited to estimate the prevalence of hypertension because recording direct blood pressure measurements was not considered primarily due to logistical and feasibility constraints, and the community-based nature of the study, which made standardized clinical measurements challenging to implement. It included four sections: sociodemographic characteristics, lifestyle factors, and preventive practices. A pilot test with five participants confirmed clarity and feasibility, with minor revisions made. Feedback obtained from the pilot study was used to refine question wording, improve comprehension, and ensure the logical flow of the instrument. Due to time and logistical constraints, a larger pilot sample was not feasible. The final questionnaire was administered face-to-face by researchers to ensure completeness of responses.

### 2.3. Methodology

Data collection was carried out at various public social venues across multiple Emirates, including shopping malls, parks, and community centers. By selecting multiple locations, the study was able to capture a diverse population of young adults from different socioeconomic backgrounds, nationalities, and educational levels. This approach was intended to ensure that the findings reflected the heterogeneity of the UAE’s young adult population and provided a holistic view of hypertension prevalence and its determinants in real-world community settings.

### 2.4. Ethical Aspects

Ethical approval was obtained from the Medical University Institutional Review Board, Ref. no. IRB-COM-STD-30-Jan-2025. Participation was entirely voluntary, and informed consent was obtained from each individual before enrollment in the study. Informed consent procedures ensured that participants were fully aware of the purpose of the study, the procedures involved, and their rights to withdraw at any point without any consequences. Confidentiality was ensured.

### 2.5. Data Management and Analysis

Data collected will be downloaded to an Excel spreadsheet. Imported into SPSS (Version 29) for statistical analysis. Findings were reported in terms of frequency and percentages, if appropriate. Chi-square test was used to assess relationships between dependent and independent variables. Significance level set at *p* ≤ 0.05.

## 3. Results

Among 1606 participants, 58.2% were <30 years and 41.8% ≥30 years. Males constituted 64.4% and females 35.6%. Most were from the African region (88.8%), single (53.7%), highly educated (90.5% graduates), and employed (84.6%).

**Figure 1:**
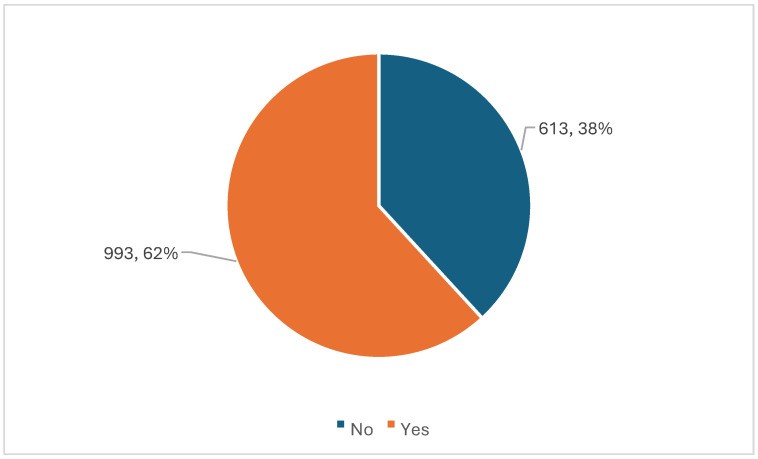
Distribution of Participants according to Self-reported History of Hypertension.

Figure 1 shows that 62% (993) of participants reported a history of hypertension, while 38% (613) did not, indicating a majority had experienced hypertension.

Only 22.1% of participants had controlled hypertension, while 77.9% remained uncontrolled. Medication use was low (17.8%), with many relying on herbal remedies (51.5%) and minimal lifestyle modification (4.9%). A high proportion reported family history of hypertension (54.2%) and chronic diseases (72.2%).

Despite 82.4% being physically active, unhealthy habits were common, including frequent fast food, salty snacks, and high caffeine intake. Most participants (85.1%) reported lifestyle changes post-diagnosis, yet gaps remained in follow-up (36.4%) and regular blood pressure monitoring (40.6%), highlighting the need for better adherence and patient education.

A significant association was found between hypertension and sociodemographic factors, as given in Table 1. Higher prevalence was observed among participants aged ≥30 years, males, African nationals, married individuals, those with graduate education or above, and employed participants (all *p* ≤ 0.04).

A strong association was observed between hypertension and family/personal health history. Participants with a family history of hypertension (74.6%) or chronic conditions (59.8%) showed higher prevalence, as did those with a personal history of chronic disease (85.2%), compared to those without (*p* = 0.001). Details are given in Table 2.

Lifestyle factors showed significant associations with hypertension. Both low and high salt intake were linked to a higher prevalence compared to moderate intake. Physical activity was protective, with lower rates among inactive individuals. Tobacco use and alcohol consumption were also strongly associated with higher hypertension prevalence (all *p* ≤ 0.002). Details are given in Table 3.

Preventive behaviors were significantly associated with hypertension. Regular blood pressure monitoring, healthcare follow-up, and lifestyle modification were linked to higher detected prevalence, reflecting increased diagnosis and awareness among these groups (*p* = 0.001). Details are given in Table 4.

The association between sociodemographic, behavioral, and clinical factors with hypertension was assessed using both bivariate and multivariate analyses.

In the crude analysis, participants aged less than 30 years had significantly higher odds of hypertension compared to those aged ≥30 years (OR = 1.30, 95% CI: 1.07–1.62, *p* = 0.007). This association remained significant after adjustment (AOR = 1.88, 95% CI: 1.41–2.49, *p* = 0.001).

Male participants were more likely to report hypertension than females (OR = 2.42, 95% CI: 1.96–2.99, *p* < 0.001), and this association persisted in the adjusted model (AOR = 2.44, 95% CI: 1.83–3.26, *p* = 0.001).

Nationality showed a strong association, with participants from the African region having significantly higher odds of hypertension compared to others (OR = 10.4, 95% CI: 6.92–15.62), which remained significant after adjustment (AOR = 7.75, 95% CI: 4.70–12.78, *p* = 0.001).

Marital status and family history of chronic conditions were not significantly associated with hypertension. Similarly, salt intake categories did not show a statistically significant association.

Higher educational status (graduate and above) was significantly associated with increased odds of hypertension (OR = 4.35, 95% CI: 3.03–6.25, *p* = 0.001), and this remained significant after adjustment (AOR = 1.90, 95% CI: 1.08–3.35, *p* = 0.02).

Employed participants had markedly higher odds of hypertension compared to unemployed individuals (OR = 12.65, 95% CI: 8.81–18.15, *p* = 0.001), which remained significant after adjustment (AOR = 8.32, 95% CI: 5.22–13.25, *p* = 0.001).

Participants with a family history of hypertension had increased odds of hypertension (OR = 2.81, 95% CI: 2.27–3.48, *p* = 0.001), which was further strengthened in the adjusted analysis (AOR = 4.89, 95% CI: 3.37–7.10, *p* = 0.001). Similarly, those with a personal history of chronic conditions had significantly higher odds of hypertension (OR = 5.16, 95% CI: 3.87–6.86, *p* = 0.001), remaining significant after adjustment (AOR = 5.71, 95% CI: 3.76–8.69, *p* = 0.001).

Behavioral factors such as tobacco use and alcohol consumption were significantly associated with hypertension. Tobacco users had higher odds (OR = 2.69, 95% CI: 2.10–3.44, *p* = 0.001), which remained significant after adjustment (AOR = 1.80, 95% CI: 1.28–2.53, *p* = 0.001). Alcohol consumption was also significantly associated (OR = 4.32, 95% CI: 3.00–6.23, *p* = 0.001), and remained significant in the adjusted model (AOR = 2.86, 95% CI: 1.77–4.64, *p* = 0.001). Although physical activity variables were significant in crude analysis, they were not included in the adjusted model. Details are given in Table 5. 

## 4. Discussion

In this study, 61.8% of participants reported hypertension—nearly double the ~28–35% prevalence reported in previous UAE studies. This higher rate may reflect increasing hypertension among young adults, sample characteristics (predominantly African nationals), and reliance on self-reported data [1,2,3,4,5].

Consistent with Alketbi et al. [3], this study found higher hypertension rates among participants aged ≥30 years and males. Similar male predominance reported in the UAE Healthy Future Study suggests that hormonal, behavioral, and occupational factors may contribute to this gender disparity. Unlike Bhagavathula et al. [1], which reported an inverse relationship between education and hypertension, this study found higher prevalence among university-educated and employed participants, possibly due to occupational stress and sedentary lifestyles. A strong association with family history aligns with the Dubai Household Survey [2] and the UAE Healthy Future Study [5], highlighting genetic and household clustering of cardiometabolic risks.

Evidence suggests that both hereditary and shared lifestyle behaviors contribute to hypertension risk among UAE residents. Lifestyle factors such as physical inactivity, smoking, and alcohol use were associated with higher hypertension rates, consistent with Shah et al. [4] and Bhagavathula et al. [1]. Conversely, physical activity appeared protective, reinforcing lifestyle modification as a key non-pharmacological strategy for hypertension prevention and control. Despite the high burden, hypertension control was poor, with only 22.1% achieving control and 17.8% on antihypertensive medication. Similar low control rates were reported by Bhagavathula et al. [1] and Shah et al. [4]. Notably, herbal medicine use (51.5%) exceeded modern therapy (43.6%), reflecting cultural preferences and possible gaps in access, trust, or adherence, as also noted by Mamdouh et al. [2].

The findings highlight an urgent public health concern, with hypertension emerging at younger ages and remaining poorly controlled. Compared with national averages below 35%, the higher prevalence indicates early onset and inadequate management. Consistent with Alketbi et al. [3] and Mezhal et al. [5], there is a need for youth-focused screening, lifestyle education, and culturally tailored interventions, including routine monitoring, physical activity promotion, and integration of traditional beliefs into prevention strategies.

Hypertension was more prevalent among males, consistent with regional and international studies, likely due to hormonal, behavioral, and lifestyle differences, as well as lower healthcare engagement among men [6,7,8]. Participants aged ≥30 years also showed higher prevalence, aligning with prior research [6,9], reflecting age-related vascular changes and the early impact of lifestyle factors during the transition from the late 20s to early 30s.

Lifestyle factors significantly influenced hypertension risk, with physical inactivity and lack of dietary modification linked to higher blood pressure. This aligns with evidence highlighting sedentary behavior, poor diet, and high salt intake as key contributors among young adults in the Gulf region [7,10]. Smoking and poor follow-up were strongly linked to uncontrolled blood pressure, consistent with evidence on vascular dysfunction. Conversely, regular monitoring and healthcare follow-up were associated with better control, highlighting the importance of lifestyle modification, patient engagement, and adherence to medical advice in the management of hypertension [7,8,9,10]. Lack of follow-up was a key barrier to hypertension control, especially among young adults who may underestimate its asymptomatic nature. Overall, hypertension risk was influenced by multiple factors, including age, gender, lifestyle, and healthcare engagement. These findings provide important UAE-specific insights and reinforce the need for early detection, education, and sustained behavioral interventions in younger populations [6,7,8,9,10].

Participants who regularly monitored their blood pressure had higher hypertension detection (71.6%) than those who did not (47.4%) (*p* = 0.001). Regular healthcare follow-up was also associated with better awareness and control (76.0% vs. 37.6%, *p* = 0.001). These findings highlight the importance of routine screening and continuous follow-up in preventing hypertension complications [11].

Participants who adopted lifestyle changes after diagnosis showed better control (80.9%) than those who did not (54.0%) (*p* = 0.001). This supports public health initiatives like the UAE National Nutrition and Salt Reduction Policy, promoting healthy diets and reduced sodium intake [12,13,14,15]. Regular physical activity was also protective, with lower hypertension prevalence among active individuals (63.9%) compared to inactive ones (48.1%) (*p* = 0.001), though many UAE youth do not meet WHO exercise recommendations. Additionally, tobacco (77.2%) and alcohol use (85.4%) were associated with significantly higher hypertension prevalence compared to non-users, reinforcing their role as key modifiable risk factors [15,16,17,18,19,20,21].

The present study identified several significant predictors of Hypertension among young adults, including younger age (<30 years), male gender, African nationality, higher education, employment status, family history of hypertension, personal history of chronic conditions, and behavioral factors such as tobacco and alcohol use. These findings are broadly consistent with emerging evidence that hypertension is increasingly prevalent in younger populations [18,22,23].

The higher odds observed among males align with recent studies indicating that young men are more likely to develop hypertension due to a combination of biological factors (e.g., hormonal influences) and behavioral risks such as smoking and alcohol consumption. Similarly, the strong association with African nationality is supported by global literature, which consistently reports higher hypertension prevalence among individuals of African origin, potentially due to genetic predisposition, salt sensitivity, and environmental influences [11,24,25,26].

Interestingly, participants aged <30 years demonstrated higher odds of hypertension, which contrasts with traditional patterns where prevalence increases with age. However, recent studies have highlighted a growing burden of hypertension in younger adults, largely driven by sedentary lifestyles, stress, unhealthy dietary habits, and early exposure to risk factors. The association with higher education and employment may reflect occupational stress, prolonged sedentary work, and lifestyle transitions commonly observed in urbanized settings [23,27].

Clinical predictors such as family history of hypertension and personal history of chronic conditions showed strong associations, consistent with existing evidence emphasizing genetic susceptibility and clustering of non-communicable diseases. Behavioral factors, particularly tobacco use and alcohol consumption, remained significant predictors after adjustment, reinforcing their well-established role in elevating blood pressure through vascular and neurohormonal mechanisms [11,24,26,28].

In contrast, variables such as marital status and salt intake were not significantly associated with hypertension in this study. This may be due to measurement limitations, self-reported data, or insufficient variability within the sample. Additionally, the lack of association with salt intake could reflect underestimation or misclassification of dietary exposure, which is commonly reported in survey-based studies. These findings highlight the multifactorial nature of hypertension among young adults and underscore the need for targeted prevention strategies focusing on modifiable behavioral risk factors, early screening, and lifestyle interventions in this age group.

This study underscores the need for targeted health promotion in the UAE, focusing on modifiable risk factors such as diet, physical inactivity, and smoking. Integrating routine blood pressure screening and education into primary care can help reduce the burden of hypertension among young adults. Future research should use longitudinal designs and culturally tailored interventions to improve lifestyle adherence and follow-up. Although this study provides valuable insights into the prevalence, risk factors, and preventive strategies of hypertension among young adults in the United Arab Emirates, several limitations should be acknowledged. First, the study utilized a cross-sectional design, which limits the ability to establish causal relationships between identified risk factors and hypertension. This research solely depended on self-reported physicians diagnosed hypertension. Future studies incorporating objective blood pressure measurements would strengthen the validity of the findings. Future studies with larger pilot samples and formal validation procedures are recommended to strengthen the tool’s psychometric properties. The study was conducted within a specific accessible population, and therefore, the sample may not accurately reflect the diverse demographic structure of the UAE. The overrepresentation of certain groups may have influenced the observed prevalence estimates and associated factors. We also recommend that future studies employ probability-based sampling methods with more representative and diverse populations to enhance external validity and allow for more generalizable conclusions.

## 5. Conclusions

This study highlights a high prevalence of Hypertension among young adults in the United Arab Emirates, driven by sociodemographic and lifestyle factors such as male gender, increasing age, family history, and unhealthy behaviors. Despite reasonable awareness, blood pressure control and medication adherence remain low, with notable reliance on herbal remedies.

The findings emphasize the need for youth-focused prevention strategies, including regular screening, lifestyle counseling, and targeted health education. Strengthening early detection and promoting sustained behavioral change are essential to reduce future cardiovascular risks.

## Figures and Tables

**Table 1:** Association between Sociodemographic Characteristics and History of Hypertension.

Sociodemographic Characteristics	Groups	History of Hypertension				*p* Value
		Yes		No		
		No.	%	No.	%	
Age group in Years	Less than 30 years	604	64.6	331	35.4	0.007
	Greater than or equal to 30 years	389	58.0	282	42.0	
Gender	Male	717	69.3	317	30.7	0.001
	Female	276	48.3	296	51.7	
Nationality	Africa Region	963	67.5	463	32.5	0.001
	Others	30	16.7	150	83.3	
Marital status	Single	517	59.9	346	40.1	0.04
	Married	476	64.1	267	35.9	
Level of Education	Below Undergraduate	46	30.1	107	69.9	0.001
	Graduate and above	947	65.2	506	34.8	
Employment status	Employed	954	70.3	404	29.7	0.001
	Unemployed	39	15.7	209	84.3	

**Table 2:** Association between Family history, personal history, and history of Hypertension.

Family History, Personal History, and the History of Hypertension	Groups	History of Hypertension				*p* Value
		Yes		No		
		No.	%	No.	%	
Family History of Hypertension	Ye	549	74.6	187	25.4	0.001
	No	444	51	426	49	
Personal History of Chronic Conditions	Yes	381	85.2	66	14.8	0.001
	No	612	52.8	547	47.2	
Family history of Chronic conditions	Yes	388	59.8	261	40.2	0.001
	No	605	63.2	352	36.8	

**Table 3:** Association between Lifestyle factors and history of Hypertension.

Lifestyle Factors	Groups	History of Hypertension				*p* Value
		Yes		No		
		No.	%	No.	%	
Salt intake	Low	318	67.5	153	32.5	0.002
	Moderate	586	58.5	416	41.5	
	High	89	66.9	44	33.1	
Physically Active	Yes	846	63.9	477	36.1	0.001
	No	136	48.1	136	48.1	
Perform physical activity	Yes	629	66.6	316	33.4	0.001
	No	364	55.1	297	44.9	
Use of Tobacco Products	Yes	355	77.2	105	22.8	0.001
	No	508	44.3	638	55.7	
Do you consume Alcohol	Yes	216	85.4	37	14.6	0.001
	No	777	57.4	576	42.6	

**Table 4:** Association between Preventive Practice and history of Hypertension.

Preventive Practice	Groups	History of Hypertension				*p* Value
		Yes		No		
Get your blood Pressure monitored regularly	Yes	677	71.6	268	28.4	0.001
	No	307	47.4	340	52.6	
Follow-up with Healthcare Providers	Yes	768	76	242	24	0.001
	No	217	37.6	360	62.4	
Modified Lifestyle after being diagnosed with Hypertension	Yes	693	80.9	164	19.1	0.001
	No	81	54	69	46	

**Table 5:** Logistic Regression for Predictors of Hypertension.

Variables	Group	Hypertension					
		Crude			Adjusted		
		OR	CI	*p*-Value	OR	CI	*p*-Value
Age Group	Less than 30 years	1.3	1.07–1.62	0.007	1.88	1.41–2.49	0.001
	Greater than or equal to 30 years	1	--	--			
Gender	Male	2.42	1.96–2.99	0.000	2.44	1.83–3.26	0.001
	Female	1	--	--			
Nationality	Africa Region	10.4	6.92–15.62		7.75	4.70–12.78	0.001
	Others	1	-	-			
Marital status	Single	1			--		
	Married	1.19	0.97–1.46	0.08	--		
Level of Education	Below Undergraduate	1					
	Graduate and above	4.35	3.03–6.25	0.001	1.90	1.08–3.35	0.02
Employment status	Employed	12.65	8.81–18.15	0.001	8.32	5.22–13.25	0.001
	Unemployed	1					
Family History of Hypertension	Yes	2.81	2.27–3.48	0.001	4.89	3.37–7.10	0.001
	No	1					
Personal History of Chronic Conditions	Yes	5.16	3.87–6.86	0.001	5.71	3.76–8.69	0.001
	No	1					
Family history of Chronic conditions	Yes	1					
	No	1.15	0.94–1.41	0.16	--		
Salt intake	Low	1.02	0.68–1.54	0.89	--		
	Moderate	0.64	0.47–1.02	0.06	--		
	High	1					
Physically Active	Yes	1.64	1.26–2.12	0.001	--		
	No	1					
Perform physical activity	Yes	1.62	1.32–1.99	0.001	--		
	No	1					
Use of Tobacco Products	Yes	2.69	2.10–3.44	0.001	1.80	1.28–2.53	0.001
	No	1					
Do you consume Alcohol	Yes	4.32	3.00–6.23	0.001	2.86	1.77–4.64	0.001
	No	1					

## Data Availability

The data related to the study is presented in the article.
